# Microcellular foams made from gliadin

**DOI:** 10.1007/s00396-014-3317-6

**Published:** 2014-06-28

**Authors:** S. Quester, M. Dahesh, R. Strey

**Affiliations:** 1 Department of Chemistry, Institute of Physical Chemistry, University of Cologne, 50939 Cologne, Germany; 2 Laboratoire Charles Coulomb UMR 5221, Université Montpellier 2, CNRS, 34095 Montpellier, France; 3 Laboratoire Charles Coulomb UMR 5221, CNRS, 34095 Montpellier, France; 4 UMR IATE, UM2-CIRAD-INRA-SupAgro, 2 Place Pierre Viala, 34070 Montpellier, France

**Keywords:** Microfoam, Nanofoam, Gliadin, Foaming, scCO_2_

## Abstract

We have generated closed-cell microcellular foams from gliadin, an abundantly available wheat storage protein. The extraction procedure of gliadin from wheat gluten, which involves only the natural solvents water and ethanol, respectively, is described with emphasis on the precipitation step of gliadin which results in a fine dispersion of mostly spherical, submicron gliadin particles composed of myriad of protein molecules. A dense packing of these particles was hydrated and subjected to an atmosphere of carbon dioxide or nitrogen in a high-pressure cell at 250 bar. Subsequent heating to temperatures close to but still below 100 °C followed by sudden expansion and simultaneous cooling resulted in closed-cell microcellular foam. The spherical gliadin templates along with the resulting foam have been analyzed by scanning electron microscope (SEM) pictures. The size distribution of the primary particles shows diameters peaked around 0.54 μm, and the final foam cell size peaks around 1.2 μm, at a porosity of about 80 %. These are the smallest foam cell sizes ever reported for gliadin. Interestingly, the cell walls of these microcellular foams are remarkably thin with thicknesses in the lower nanometer range, thus nourishing the hope to be able to reach gliadin nanofoam.

## Introduction

Gliadin, a heterogeneous mixture of single-chained or monomeric proteins with a molecular mass between roughly 25 and 75 kDa, represents about the half of the seed storage proteins of wheat kernels [1–3]. Based on their electrophoretic mobility at low pH, α-/β-, γ- and ω-gliadins are distinguished [4]. Their mass varies from 25 to 35 kDa for the α-type, 30–35 kDa for the β-type, 35–40 kDa for the γ-type, and 55–75 kDa for the ω-type [5]. The α- and β-gliadins are closely related and therefore often typed as α-gliadin. The sulfur-rich α-/β- and γ-gliadins show a similar primary structure of polypeptides with cysteine residues in the native state connected by inter-chain disulfide bonds, whereas the sulfur-poor ω-gliadins, which lack cysteine, cannot form cross-links [2, 6]. With their intramolecular disulfide bonds, gliadins contribute to the viscous nature of dough [7]. They are characterized by a low electrostatic charge density and a poor solubility in aqueous salt solutions but a good solubility in alcohol water mixtures [8]. Besides the monomeric gliadin, wheat storage proteins also contain polymeric proteins, so-called glutenins, with masses from about 80 to several thousand kilodaltons. The elastic and strength properties of dough, for example, are ascribed to the glutenin polymers, whereas the gliadins are believed to act as plasticizers that weaken the interactions between the glutenin chains leading thereby to an increase of the dough viscosity [9]. The terms mono and polymeric refer to the quaternary structure of the protein. Referring to the high proportions of glutamine and proline, especially in the central hydrophilic domain [9], these proteins are classified as “prolamins” [9].

Wheat storage proteins constitute an interesting green material from renewable resources for various applications, not only in the food sector. Due to the unique structural and functional properties of gluten, it has experienced attention not only in baking processes. It is known from Banc et al. that gliadin shows surface-active properties [10], one characteristic feature which is utilized to stabilize gas cells during bread making [9]. Its viscoelastic properties and low water solubility are interesting features for non-food applications [6, 11, 12]. The different gluten proteins and mixtures thereof with their different characteristics might be utilized to tailor foams with special properties. Blomfeldt et al. proposed wheat gluten as an interesting alternative, especially for making polymer foams [12]. In the non-food, non-bio world, the quest for foams of nanoscopic sizes and low densities continues, as such so-called nanofoams constitute a promising nanoinsulation material [13]. It could, e.g., be used to better insulate houses and refrigerators. Of course, a clean production of nanofoams from biomaterial would greatly enhance the green aspect of energy saving by insulation. In any case, already, microfoam from gliadin is an interesting alternative to conventional polymer foams.

The structure of the gliadin proteins, which form together with the polymeric glutenin proteins some of the most complex network structures in nature [14], is itself already complex due to the heterogeneity of the individual proteins and the wide range of their molecular weights.

## State of the art

The excellent foaming properties of gliadin [9] are known for decades. McDonald and Pence described in 1961 stable foams similar to those prepared from egg white resulting from whipped aqueous solutions of gliadin [15]. Compared to egg white, gelatin, soy, and milk proteins, gliadin exhibited superior performance at no increase or even reduction in cost. In 1977, Mita et al. studied the stability of gluten foams and found the mechanical properties of the surface to be responsible for the foam stability [16, 17]. Hernandez-Munoz et al. reported about the excellent film-forming properties and the long-term stability which qualifies gliadin as a good candidate for the production of sustainable films and coatings, e.g., for food packaging [18]. Thewissen et al. emphasized the viscoelastic properties accessible by hydration of the gliadin as its predominant feature [19–21]. In 2010, Blomfeldt et al. termed gluten a “fascinatingly versatile material” which can be processed with conventional polymer processing techniques into various 3D forms [22]. Two years later, these authors reported freeze-dried foams from gliadin and gluten-rich fractions of gliadin as well as from gluten with mean pore size diameters between 20 and 73 μm [23].

## Experimental

### Materials

Gliadin was extracted from commercial wheat gluten (courtesy of Tereos Syral, France). The gliadin extraction method was adapted from Boire et al. [3]. Briefly, 100 g of wheat gluten were dispersed in 1 L of an ethanol/water mixture 50 % (*v*/*v*) and stirred overnight at 20 °C. The supernatant recovered after centrifugation for 30 min at 20 °C, and 15,000 g was cooled to and kept overnight at 4 °C which resulted in phase separation. The upper phase which contains almost only gliadin was separated and kept under a fume hood in order to evaporate the ethanol before freeze-drying. The gliadin yield was about 18 g.

**Fig. 1:**
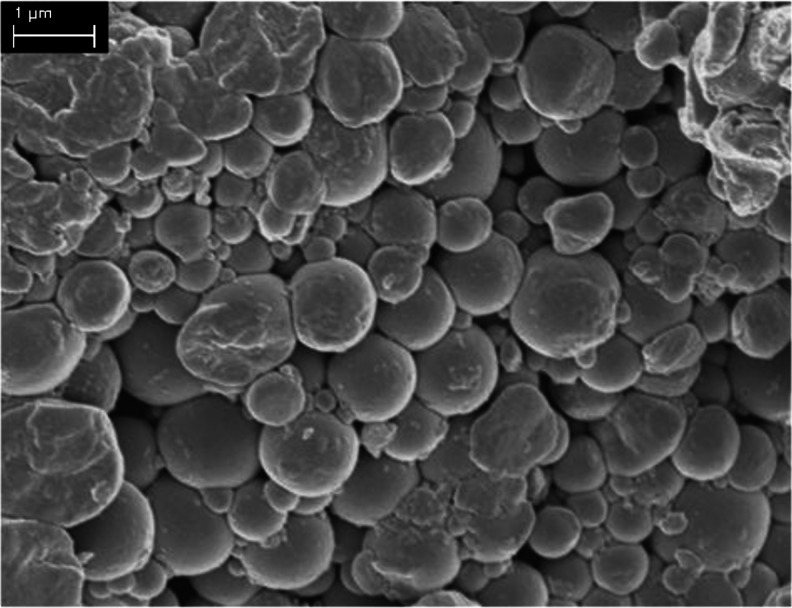
SEM picture of the gliadin primary particles

In Fig. 1, the structure of the original dried gliadin powder is shown featuring a characteristic collection of spherical particles, although also, some larger particles and irregular shaped blocks are visible. Analyzing the predominantly spherical particles, we find the sizes to vary from roughly 100 nm to 1 μm.

### Preparation of starting material

For further processing, the gliadin material as seen in Fig. 1 was contacted with water in the following manner. Under gentle stirring, purified water (filtered through a Millipore membrane) was dropwise added to the dry gliadin powder at room temperature. Thereby, the mixture became successively more deformable and flowable, but still a viscous paste. To avoid the intake of air bubbles, stirring was performed very carefully just to achieve a homogenous state. The water content was 33 wt%.

**Fig. 2:**
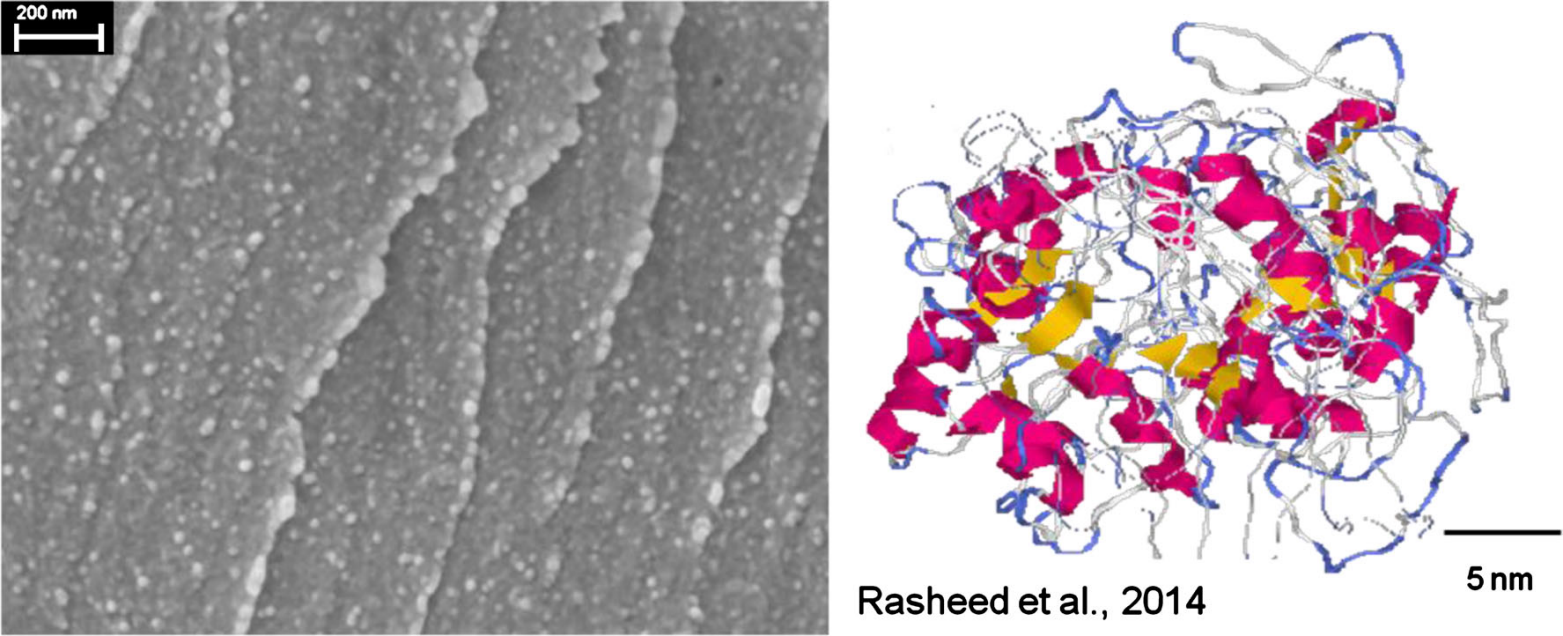
SEM picture of the cross-section of the dried hydrated gliadin (*left*). A predicted model structure of unmodified gliadin (*right*) (provided by and reproduced with permission of the authors, Rasheed et al. [14])

Scanning electron microscope (SEM) pictures of the highly viscous mass after 24 h in a vacuum desiccator show a very homogenous structured material of close-packed spheres with diameters between roughly 10 and 30 nm (Fig. 2, left). The individual protein spheres can almost be resolved and appear as visible as points. To get a feel for the structure of the proteins, the recently proposed structure model of unmodified gliadin (α/β, γ, ω) (Rasheed et al. [14]) is shown on Fig. 2, right.

### Preparation of foam

A portion of the viscous hydrated gliadin starting material was cut with a scalpel, weighed in a sample pan, and placed in a high-pressure cell. The cell consists of a 4-cm-long, hollow sapphire cylinder standing upright in a water bath. The inner bore is 1 cm, the wall thickness 1 cm. The pressure can be regulated by pressing down a tightly fitting piston into the inner bore. The pressure is recorded electronically. An external valve permits a pressure release with an initial d*p*/d*t* = 100 bar s^−1^ or less. The cell was filled with fluid CO_2_ at room temperature at a pressure of 72 bar and was then placed in a water bath at a temperature of 95 °C adjusting the rising pressure to 250 bar. Ninety-five degree Celsius were chosen to stay below the ordinary boiling point of water. After 30 min, a sudden expansion to atmospheric pressure was performed while the high-pressure cell was cooled in a second water bath to 25 °C within 2 min. The sample was dried in a vacuum desiccator for at least 1 day and was examined under a SEM.

**Fig. 3:**
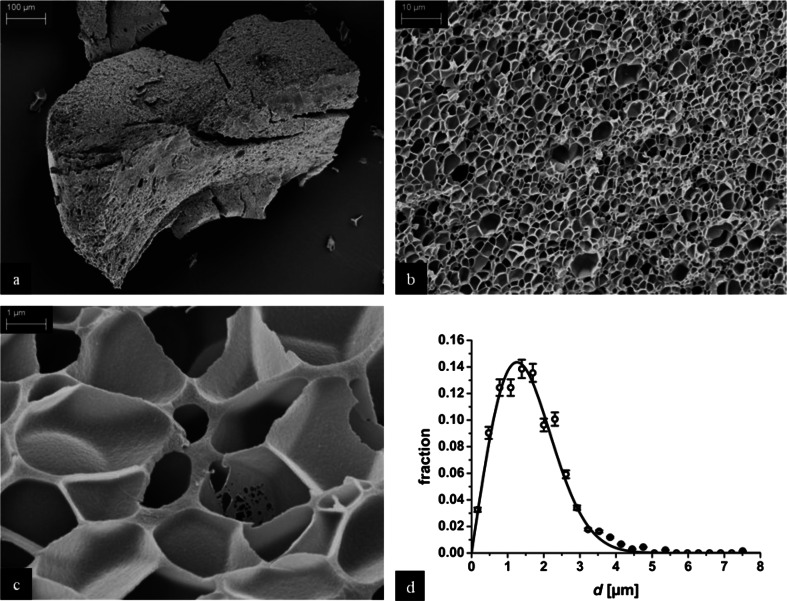
**a**–**c** SEM pictures of the resulting foam after the hydrated gliadin was soaked with fluid CO_2_ at room temperature and heated to 95 °C while the pressure was adjusted to 250 bar and held for 30 min followed by sudden expansion and cooling to 25 °C. **d** Cell size distribution

With no further additive (e.g., a cross-linking agent), just from the hydrated gliadin, a foam with mean cell diameters in the lower micrometer range is obtained by the procedure described above. Figure 3 displays the foam, while Fig. 3a demonstrates that the whole material is foamed and that the cells distribution is relatively homogenous. The next higher magnification in Fig. 3b shows the even distribution of the foam cells. The highest magnification in Fig. 3c clearly illustrates the individual cells of the smallest gliadin foam reported so far. Interestingly, the membranes of the foam windows are in some areas extremely thin formed by presumably only a single layer of gliadin molecules.

To evaluate the cell size distribution, the largest extensions of 1,350 individual cells in Fig. 3b were measured, referred to as *d*. The diagram in Fig. 3d shows the foam cell distribution along with a (modified Gaussian) fit function (*f* (*d*) = *a d* exp (−(*d* − *d*_max_)^2^ / (2σ^2^)), *a* = 0.175 μm^−1^, σ = 0.14 μm, *d*_max_ = 1.2 μm). From Fig. 3c, we have evaluated the number density of foam bubbles according to *N*_f_ = (*nM* ^2^ / *A*)^3/2^ = 3,6 · 10^11^ cm^−3^ [24, eq. 1], where *n* was the number of cells in, *M* the magnification factor, and *A* the area of the micrograph, respectively. This compares favorably with the estimate for the number density *N*_f_ = *d*_max_^−3^ = 5.8 · 10^11^ cm^−3^ from the maximum of the cell size distribution *d*_max_ in Fig. 3c. About 20 % of the foam is solid material; thus, the porosity is 0.8.

The tuning of the rheological characteristics of the material by degree of hydration and temperature [25] facilitates disulfide/sulfhydryl interchange reactions, which can end up in the formation of intermolecular disulfide bonds between the gliadin units providing stability to the structure. During the expansion process, the interface experiences enormous stress. Therefore, the relatively homogenous structure and size distribution of the closed cells is quite surprising, indicating once more the unique features of this material. The protein molecules are able to perform hydrophobic interactions and—probably more importantly—to form disulfide bridges which enable the formation of network structures [26]. This process is known from processing of gliadin films. For instance, it was recently reported from thermo-processing of gliadin resin [21]. As already mentioned, the α-/β- and γ-gliadins contain significant levels of disulfide or sulfhydryl groups that potentially can be involved in the formation of new disulfide bonds, while the ω-gliadins are deficient and in some cases completely lacking of sulfur-containing amino acids and therefore cannot participate in the formation of disulfide bonds [27, 28].

A reduction of the size of the foam bubbles by a factor of about ten appears reachable, possibly by a reduction of the size of the primary particles which are to be hydrated and thereby of the accompanying voids. We presume that the morphology and mean cell size of the final foam is the result of the interplay between the degree of hydration, especially a homogenous hydration of the sample, rheological characteristics which significantly depend on temperature, pressure, and time, the nature of the blowing agent, and the temporal evolution of temperature during the expansion. This very complex parameter space has not yet been explored. Currently, we are performing a systematic variation of the parameters in order to develop an understanding of the influence of the different parameters with regard to the foam morphology including the cell size distribution, the wall thickness, and the porosity. Presently, we presume increasing pressure and temperature while decreasing the rate of pressure release should result in an increase of the number density of cells accompanied by simultaneous decrease of the thickness of the cell walls. The foams obtained so far were prepared just from gliadin proteins plasticized by water without further additives. They appear to be quite elastic and irreversibly deformable, but are also brittle to a certain degree. The foam characteristics can further be tuned by addition of plasticizer such as glycerol [9] and stabilizers or by other substances promoting polymerization. By this, the mechanical properties can be tuned to achieve, for example, flexibility which might be required for packing materials or other applications [29]. Mangavel et al. addressed to the mechanical properties of gliadin films and their improvement and found a significant rise of the tensile strength as well as a decrease of elongation at break of the films for increased drying temperatures along with a decrease of the water solubility (indicating some network strengthening induced by thermal treatment) [30].

## Conclusion

Closed-cell gliadin foams with very thin membranes of only a few nanometers were obtained in the first foaming experiments. The mean diameter of the foam cells is only slightly larger than 1 micron. These are the smallest foam cell sizes ever reported for gliadin foams. The porosity of 0.8 is—for a first set of experiments—already quite high. We are confident that it can further be increased towards unity by increasing the pressure drop, optimizing the pressure drop rate, the temperature, and the degree of hydration. Increasing pressure and temperature while decreasing the pressure drop rate will increase the number density of bubbles while simultaneously decreasing the cell wall thickness. The brittleness of the foams might be reduced by additives. The foams prepared so far exhibit pore sizes in the lower micrometer regime, presumably as a result of coalescence or Ostwald ripening. We presume that the morphology and mean cell size of the final foam is the result of the interplay between the size and the packing of the primary particles, the degree of hydration, temperature, pressure, nature of the blowing agent, rheological parameters, and time. This complex parameter space has not yet been explored. Efforts towards this end are underway to provide a useful guide towards nanocellular foams from genuine biomaterial. The procedure reported is simple and low cost, thus lending itself to a technical realization.

## References

[1] Krejci L, Svedberg T (1934). The ultracentrifugal study of gliadin. J Am Chem Soc.

[2] Wieser H (2007). Chemistry of gluten proteins. Food Microbiol.

[3] Boire A, Menut P, Morel M-H, Sanchez C (2013). Phase behaviour of a wheat protein isolate. Soft Matter.

[4] Woychick JH, Boundy JA, Dimler RJ (1961). Starch gel electrophoresis of wheat gluten proteins with concentrated urea. Arch Biochem Biophys.

[5] Ezpeleta I, Irache JM, Stainmesse S (1996). Gliadin nanoparticles for the controlled release of all-trans-retinoic acid. Int J Pharm.

[6] Lagrain B, Goderis B, Brijs K, Delcour JA (2010). Molecular basis of processing wheat gluten toward biobased materials. Biomacromolecules.

[7] Ang S, Kogulanathan J, Morris GA (2010). Structure and heterogeneity of gliadin: a hydrodynamic evaluation. Eur Biophys J.

[8] Anderson OD, Litts JC, Gamier M, Greene FC (1984). Nucleic acid sequence and chromosome assignment of a wheat storage protein gene. Nucleic Acids Res.

[9] Thewissen BG, Celus I, Brijs K, Delcour JA (2011). Foaming properties of wheat gliadin. J Agric Food Chem.

[10] Banc A, Desbat B, Renard D (2007). Structure and orientation changes of omega- and gamma-gliadins at the air-water interface: a PM-IRRAS spectroscopy and Brewster angle microscopy study. Langmuir.

[11] Bietz JA, Lookhart GL (1996). Properties and non-food potential of gluten. Cereal Foods World.

[12] Blomfeldt TOJ, Kuktaite R, Johansson E, Hedenqvist MS (2011). Mechanical properties and network structure of wheat gluten foams. Biomacromolecules.

[13] Jelle BP, Gustavsen A, Baetens R (2010). The path to the high performance thermal building insulation materials and solutions of tomorrow. J Build Phys.

[14] Rasheed F, Newson WR, Plivelic TS (2014). Structural architecture and solubility of native and modified gliadin and glutenin proteins: non-crystalline molecular and atomic organization. RSC Adv.

[15] McDonald CE, Pence JW (1961). Wheat gliadin in foams for food products. Food Technol.

[16] Mita T, Nikai K, Hiraoka T, Matsuo S, Matsumoto H (1977). Physicochemical studies on wheat protein foams. J Colloid Interface Sci.

[17] Mita T, Ishida E, Matsumoto H (1978). Physicochemical studies on wheat protein foams II. Relationship between bubble size and stability of foams prepared with gluten and gluten components. J Colloid Interface Sci.

[18] Thewissen BG, Celus I, Brijs K, Delcour JA (2011). Foaming properties of tryptic gliadin hydrolysate peptide fractions. Food Chem.

[19] Hernández-Muñoz P, Hernández RJ (2001) Glutenin and gliadin films from wheat gluten: preparation and properties. Pap. Present. IFT Annu. Meet. New Orleans, Lousiana

[20] Hernández-Muñoz P, Kanavouras A, Ng PKW, Gavara R (2003). Development and characterization of biodegradable films made from wheat gluten protein fractions. J Agric Food Chem.

[21] Balaguer MP, Gomez-Estaca J, Cerisuelo JP (2014). Effect of thermo-pressing temperature on the functional properties of bioplastics made from a renewable wheat gliadin resin. LWT Food Sci Technol.

[22] Blomfeldt TOJ, Olsson RT, Menon M (2010). Novel foams based on freeze-dried renewable vital wheat gluten. Macromol Mater Eng.

[23] Blomfeldt TOJ, Kuktaite R, Plivelic TS (2012). Novel freeze-dried foams from glutenin- and gliadin-rich fractions. RSC Adv.

[24] Kumar V, Suh NP (1990). A process for making microcellular thermoplastic parts. Polym Eng Sci.

[25] Madeka H, Kokini JL (1994). Changes in rheological properties of gliadin as a function of temperature and moisture: development of a state diagram. J Food Eng.

[26] Örnebro J, Nylander T, Eliasson A-C (2000). Critical review—interfacial behaviour of wheat proteins. J Cereal Sci.

[27] Booth MR, Ewart JA (1968). Studies on four components of wheat gliadins. Biochim Biophys Acta.

[28] Schofield JD, Bottomley RC, Timms MF, Booth MR (1983). The effect of heat on wheat gluten and the involvement of sulphydryl-disulphide interchange reactions. J Cereal Sci.

[29] Gibson LJ, Ashby MF (1999) Cellular solids: structure and properties

[30] Mangavel C, Barbot J, Popineau Y, Gue J (2001) Evolution of wheat gliadins conformation during film formation. Cambridge University Press, UK 10.1021/jf0009899 11262042

